# Electric field enhancement and far-field radiation pattern of the nanoantenna with concentric rings

**DOI:** 10.1186/1556-276X-9-681

**Published:** 2014-12-17

**Authors:** Shih-Wen Chen, Yi-Han Huang, Bo-Kai Chao, Chun-Hway Hsueh, Jia-Han Li

**Affiliations:** Department of Engineering Science and Ocean Engineering, National Taiwan University, Taipei, 10617 Taiwan; Department of Materials Science and Engineering, National Taiwan University, Taipei, 10617 Taiwan

**Keywords:** Plasmonics, Nanoantennas, Far-field radiation pattern, Electric field enhancement, Surface-enhanced Raman scattering

## Abstract

The optical antennas have the potential in various applications because of their field enhancement and directivity control. The directivity of a dipole antenna can be improved by directivity-enhanced Raman scattering structure, which is a combination of a dipole antenna and a ring reflector layer on a ground plane. The concentric rings can collect the light into the center hole. Depending upon the geometry of the antenna inside the hole, different electric field enhancements can be achieved. In this paper, we propose to combine the concentric rings with the directivity-enhanced Raman scattering structure in order to study its electric field enhancement and the far-field radiation pattern by finite-difference time-domain simulations. Compared with the structure without the concentric rings over the ground plane, it is found that our proposed structure can obtain stronger electric field enhancements and narrower radiation beams because the gold rings can help to couple the light into the nanoantenna and they also scatter light into the far field and modify the far-field radiation pattern. The designed structures were fabricated and the chemical molecules of thiophenol were attached on the structures for surface-enhanced Raman scattering (SERS) measurements. The measured results show that the structure with concentric rings can have stronger SERS signals. The effects of the dielectric layer thickness in our proposed structure on the near-field enhancements and far-field radiation are also investigated. The proposed structure can be useful for several nanoantenna applications, such as sensing or detecting.

## Background

Surface-enhanced Raman scattering (SERS) has an important application of surface plasmons, which are the excitations of free electrons driven by the electromagnetic wave and confined at the interface between metal and dielectric material [1–3]. The apertures and corrugated structures can be used to excite the oscillated surface waves with large electric fields at the resonance frequencies. Due to the dependence of the Raman signal on the fourth power of the electric fields, SERS is sensitive to the geometric structure and is useful to detect the molecules [4–6]. Therefore, different approaches have been developed to obtain the rough surface or have tiny antennas for SERS substrate fabrications [7–10]. The optical antenna in nanoscale plays a major role on nanophotonics nowadays because of its field enhancement and directivity control. Similar to radio antennas, a ground plate adopted under the nanoantenna plays a role as a mirror, and the maximum electric field can be obtained when the matching condition of the quality factor is satisfied [11]. The directivity of a dipole antenna was improved by directivity-enhanced Raman scattering (DERS) structure, which is a combination of a dipole antenna and a ring-reflector layer on a ground plane [12]. Instead of using the ring reflector layer, the periodic stripes [13] and the periodic rings [14] have also been used to enhance the electric field coupling between the structures and nanoantennas which boost the electric fields at the gaps of nanoantennas. Moreover, the meta-surface supports high-efficiency anomalous reflections [15], and the nanobumps generate three-dimensional scattering [16] on a ground plane.

It was found that two kinds of resonance modes could exist in the structure that consists of a grating metal thin film, a sandwiched dielectric material, and a metal ground [17]. One mode depends on the grating period and the other mode results from Fabry-Pérot-like resonance. In this paper, we adopted the concentric rings as the grating structures on the dielectric layer over the metallic ground and studied their near-field enhancements and far-field radiation patterns. The concentric rings have often been used for focusing applications. When the concentric rings are formed on the metal surface of a single layer, a strong transmission is obtained through the structure called the bull’s eye structure which has been described extensively elsewhere [18–22]. It collects the light into the center hole, and the different shapes of the hole would result in different electric field enhancement from this light collection. For example, an elliptical aperture [23] and a hexagonal aperture [24] surrounded by polygonal segmented grooves are strongly polarization dependent. The transmission can be enhanced by cutting the bull’s eye antenna; i.e., a split bull’s eye structure [25]. A bowtie aperture [26] and a pair of fan-rod optical antenna [27] can achieve stronger electric fields by integrating with the bull’s eye structure. The bull’s eye structure has also been applied to control the fluorescence beams [28, 29].

Inspired by research of the bull’s eye structure, a question is hence raised as to whether the electric field can be enhanced and the beam light can be controlled if the concentric rings and dipole nanoantenna are put upon a dielectric material over the ground plane. To the best of our knowledge, this kind of structure has not yet been studied. Thus, in this paper, we propose to study a novel SERS substrate that combines the concentric rings and dipole antenna upon a dielectric material over the ground plane. Both the near-field enhancements and the far-field radiation patterns of our proposed SERS substrate are studied, and their physical mechanism is investigated. In addition, the sandwich-like structure with and without the concentric rings are fabricated, and their SERS signals are measured.

## Methods

**Figure 1:**
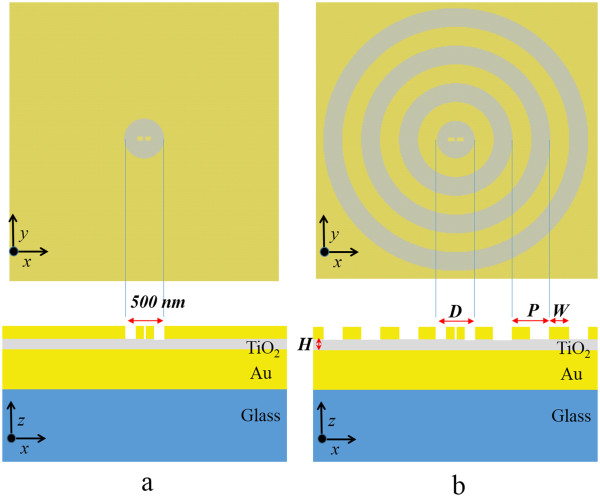
**Schematic diagrams of studied structures. (a)** the DERS structure and **(b)** the structure with concentric rings. The upper subfigure is the top view and the lower subfigure is the cross-section view. The dipole antenna in the center of the upper subfigure is 130-nm long, 45-nm wide, and 50-nm high with a 20-nm gap. The corrugated structure is placed on TiO_2_ spacer over the gold ground with a glass substrate. The diameter of the center groove is fixed at 500 nm in the DERS structure, and it is indicted as *D* in the structure-added concentric rings. The periodicity and width of the grating rings in **(b)** are denoted as *P* and *W*. The thickness of TiO_2_ spacer is 40 nm in the DERS structure in **(a)**, and it is indicated as *H* in the structure with concentric rings in **(b)**. The thickness of the gold layer atop the glass is 150 nm.

To study the near-field enhancement and the far-field radiation for the combinations of the concentric rings and the DERS structure, the parameters of the DERS structure studied in a previous work by A. Ahmed and R. Gordon [12] are adopted in our simulations. The geometry of the DERS structure is shown in Figure 1a, where the upper subfigure is the top view and the lower subfigure is the cross-section view. According to Chon et al. [5], it was designed for enhancing Raman signals with excitation laser wavelength of *λ* = 785 nm and Stokes shifted scattered radiation at *λ* = 880 nm. All parameters of the structure are set to achieve the strong surface-enhanced Raman scattering signals at the mean wavelength of *λ* = 840 nm. The dipole antenna in the center of the upper subfigure in Figure 1a is 130-nm long, 45-nm wide, and 50-nm high with a 20-nm gap, and the diameter of the central groove is 500 nm as denoted in the lower subfigure in Figure 1a, where the thicknesses of TiO_2_ spacer and Au ground plane are 40 and 150 nm, respectively. The combination of the DERS and the concentric rings structure we proposed is plotted in Figure 1b which contains several periodic gold rings and has the dipole antenna of the same size as the DERS structure. The periodicity and the width of the gold rings are denoted as *P* and *W*, respectively. The diameter of the center groove is defined as *D*. The height of the gold rings is 50 nm and the thicknesses of TiO_2_ spacer is indicated as *H*. The structure in Figure 1b can be considered as adding the periodic gold rings with the flexibility of the diameter of the center groove to the structure shown in Figure 1a. It can also be viewed as the structure of putting a nanoantenna in the center of the concentric rings. It has been shown that the resonance depends on the period of grating, and the maximum intensity can be obtained when *W* = *P*/2 [19]. Hence, the geometric parameters of our proposed structure shown in Figure 1b are set as *D* = *P* and *W* = *P*/2. We assume that there are three gold rings for simplification and simulate several cases with different periodicities of rings in our study. The Lumerical FDTD Solutions [30], a commercial software based on the finite-difference time-domain (FDTD) method, is used to simulate the near-field intensities and far-field radiations of the structures. For the near-field calculations, the incident wave is *x* polarized with the wavelength from 500 to 950 nm illuminated in the - *z* direction in Figure 1. Because the structure is symmetric, the boundary conditions at *y* = 0 and *x =* 0 are set as symmetric and anti-symmetric boundary conditions, respectively. The boundary conditions at the other sides are set as perfectly matched layer (PML) boundary conditions. Thus, the simulation domain 5,000 × 5,000 × 2,000 nm is reduced to a quarter region. A uniform mesh of 2 × 2 × 2 nm is adopted in the region of the dipole antenna 130 × 45 × 50 nm, and the non-uniform meshes are applied in the rest regions to save computation time with sufficient numerical accuracy. The time step, 3.7936 attoseconds for this mesh case, is to satisfy the numerical stability. It is worth studying the far-field radiation patterns as the nanoantenna is excited. Because the large field enhancements occurred in the gap region of the nanoantenna, an electric dipole source is placed at the feed gap of the dipole antenna to simulate the far-field radiations. The refractive indexes of gold, TiO_2_, and glass are adopted from the experiment data [31–33].

## Results and discussion

**Figure 2:**
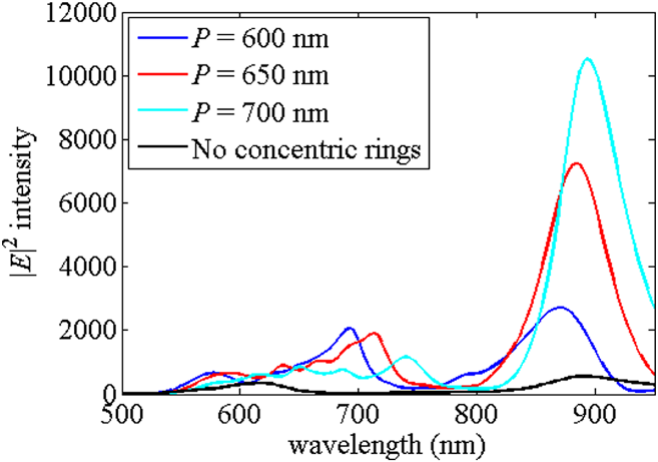
**Electric field intensity verse wavelength for the structures with and without the concentric rings.** The field intensities at the middle of the gap of the DERS structure with and without the concentric rings, where the black, blue, red, and light blue lines represent the structure with and without concentric rings with periodicity of ring *P* = 600 nm, *P* = 650 nm, and *P* = 700 nm, respectively.

For the applications in detecting molecules, the strong localized electric field can be helpful for Raman signals. Usually, the strongest electric field is near the corner of metal; however, the selected point at the middle of the gap can be treated as a fair reference. Figure 2 shows the electric field intensities |*E*|^2^ at the middle of the gap for the structures shown in Figure 1. The black line in Figure 2 represents the normalized electric field intensities |*E*|^2^ at the middle of the gap of the DERS structure in Figure 1a, and it has two peaks at *λ* = 617 nm and *λ* = 880 nm, respectively. The blue, red, and light blue lines indicate the normalized electric field intensities |*E*|^2^ at the middle of the gap of the dipole antenna with concentric rings structure for periodicity of the ring *P* = 600 nm, *P* = 650 nm, and *P* = 700 nm, respectively. It shows that the maximum intensity occurs for the case of *P* = 700 nm. However, if the excitation laser wavelength is 785 nm and detected molecule causes the Stokes shifted scattered radiation at wavelength of 880 nm, the better choice of electric field intensity enhancement from *λ* = 785 nm to *λ* = 880 nm is for the case with periodicity of *P* = 650 nm in Figure 2. For the case without concentric rings structure, the two peaks for the black line in Figure 2 occur at *λ =* 617 nm and *λ =* 880 nm. They are the resonant points when the interaction of the dipole antenna and the ground plane has the localized maximum field intensity; i.e., the transmission light reflects back by the ground plane rising the localized surface plasmons of the dipole antenna. At the maximum peaks around *λ* = 880 nm, the black line of the DERS structure has 550 times enhanced field intensity and the red line with concentric rings of periodicity *P* = 650 nm has 7,254 times enhanced electric field intensity. This is because the resonances of concentric rings structure and the DERS structure appear at the nearby wavelengths. The surface wave generated by the concentrated rings coupling into the dipole antenna which interacts with the ground plane produces the strong electric field at the center of the dipole antenna. The peaks of the red line with concentric rings of periodicity *P* = 650 nm occur around *λ =* 550 to 750 nm in Figure 2. They are the resonance peaks of the concentric rings structure, and they shift to longer wavelength when the periodicity of ring increases. According to the previous research without the ground plane [20], the maximum transmission was predicted to occur around a wavelength slightly larger than the period, *P* = 650 nm, of the concentric rings structure. Although these peaks enhance the electric field intensity in the gap of the dipole antenna, they do not overlap the resonant peaks of the DERS structure. As a result, their intensities are less than the intensity at *λ =* 880 nm.

**Figure 3:**
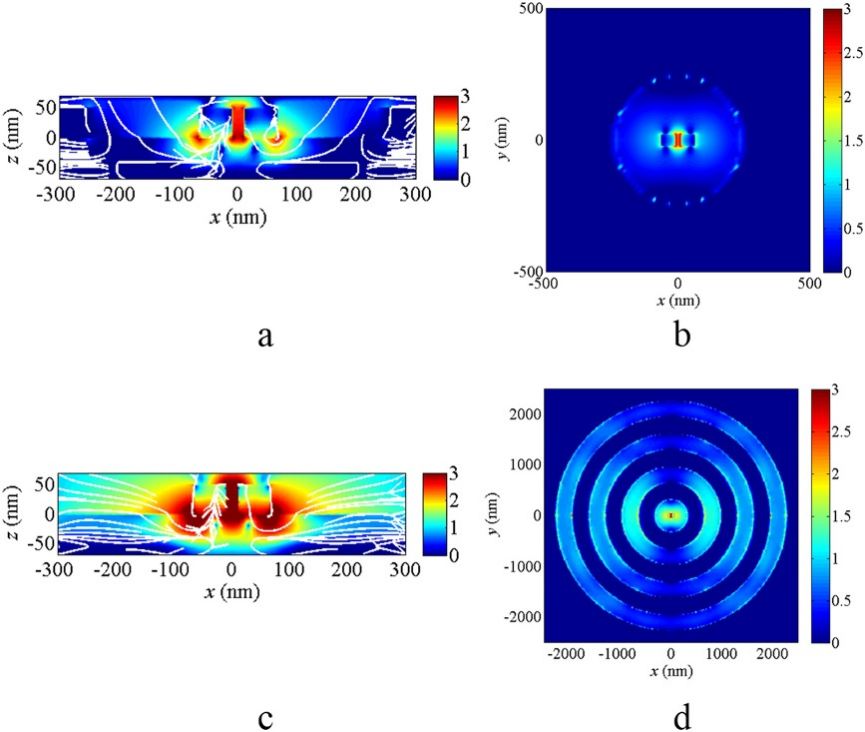
**Near-field intensities for the structures with and without the concentric rings. (a)** The cross-section view and **(b)** the top view of the electric field distribution of DESR structure at *λ* = 880 nm and **(c)** the cross-section view and **(d)** the top view of the electric field distribution of DESR structure with concentric rings at *λ* = 880 nm. The white lines are stream lines of the Poynting vectors. The scale bars are in logarithmic scale and limited to 0 to 3. The maximum field intensity of **(a)**, **(b)**, **(c)**, and **(d)** are 10^3.37^, 10^2.94^, 10^4.50^, and 10^4.08^ times of the incident light intensity. The illuminated light is the plane wave propagated from the top to bottom; i.e., in - *z* direction.

The near-field distributions of DERS structure with and without the concentric rings at *λ* = 880 nm are shown in Figure 3 with a logarithmic scale. For the easy reading of the electric field intensities, the logarithmic scale bar in Figure 3 is limited to 0 to 3. Figure 3a,b shows the electric field intensities of the cross-section view and top view, respectively, for the DERS structure without concentric rings, while Figure 3c,d is for the DERS structure with concentric rings. The white lines in Figure 3a,c shows the stream slices of the Poynting vectors. The electric fields are confined in the dipole gap and the strongest electric field intensity appears at the bottom corner of the dipole antenna, where the TiO_2_ spacer is connected. Special rotational power flows are found at the most red regions with strong electric field intensity. The concentric rings can help to couple more light into the nanoantenna for some specific wavelength, such as *λ* = 880 nm in our case. The more stream slices flow into the gap of the dipole antenna in Figure 3c as compared to the case in Figure 3a where the stream slices flow backward of the dipole antenna and then goes under the top gold layer. The inner diameter of the first gold ring in Figure 3c,d should be chosen carefully because it can affect the coupling efficiency for the electric field from the concentric rings into the nanoantenna. It is also found that there are some strong field intensities around the rings. This is because of the light coupling between the gold rings, and it can help the field coupling efficiency to the nanoantenna but it can also affect the far-field radiation pattern.

**Figure 4:**
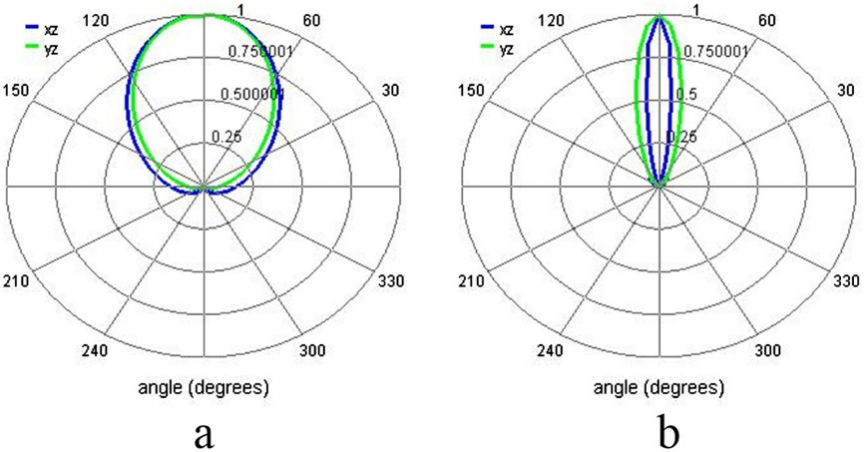
**Far-field radiation patterns for the structures with and without the concentric rings. (a)** The DERS structure and **(b)** the structure with concentric rings at *λ* = 880 nm. The radiation pattern is calculated from the simulations by placing an *x*-polarized electric dipole source at the feed gap of the dipole antenna.

To study the far-field radiation pattern by the nanoantenna in the DESR structure with and without concentric rings, we assume that an *x*-polarized electric dipole source is placed at the feed gap of the dipole antenna and calculate the far-field radiations in our simulations. Figure 4a,b shows the far-field radiation pattern for the DERS structure without and with concentric rings, respectively, at *λ* = 880 nm. It is obvious that the radiation beam is narrower for the case with concentric rings in Figure 4b than the case without concentric rings in Figure 4a. The narrower far-field radiation beam can be explained by the contributions of the gold rings which can also scatter light into far field and modify the far-field radiation pattern. It can be helpful for some applications which need to control the light beam scattering directivity, for example, the molecule detection by surface-enhanced Raman scattering.

**Figure 5:**
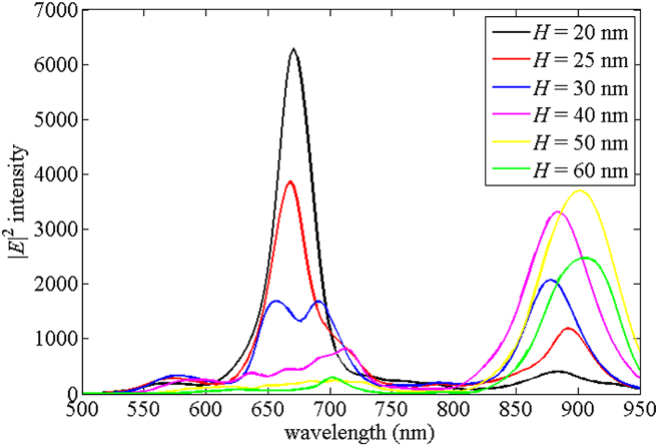
**Electric field intensity verse wavelength for the structures with concentric rings for different dielectric layer thickness.** The field intensities at the middle of the gap of the DERS structure with concentric rings with periodicity of ring *P* = 650 nm and dielectric layer thicknesses of 20, 25, 30, 40, 50, and 60 nm.

To understand the effects of the dielectric layer thickness on the near-field enhancements and far-field radiation, Figure 5 shows the simulation results of the electric field at the center of the nanoantenna for the DERS structure with concentric rings with different dielectric layer thicknesses ranging from *H* = 20 nm to *H* = 60 nm. The periodicity of *P* = 650 nm is fixed. The peaks between *λ* = 850 nm and *λ* = 950 nm shift when the dielectric layer thickness is changed. It is because the light coupling efficiency from the gold rings to the nanoantenna can be affected by the dielectric layer thickness. The peaks between *λ* = 600 nm and *λ* = 750 nm are strongly dependent on the dielectric material thickness, and the field enhancement of these peaks becomes larger as the dielectric layer thickness is smaller. The electric field in the dielectric layer can be interfered between the gold rings and bottom gold layer because the light can go back and forth between them. Thus, the large field enhancement can be achieved as the constructive interference happens by choosing a suitable layer thickness, such as the case with *H* = 20 nm in Figure 5.

**Figure 6:**
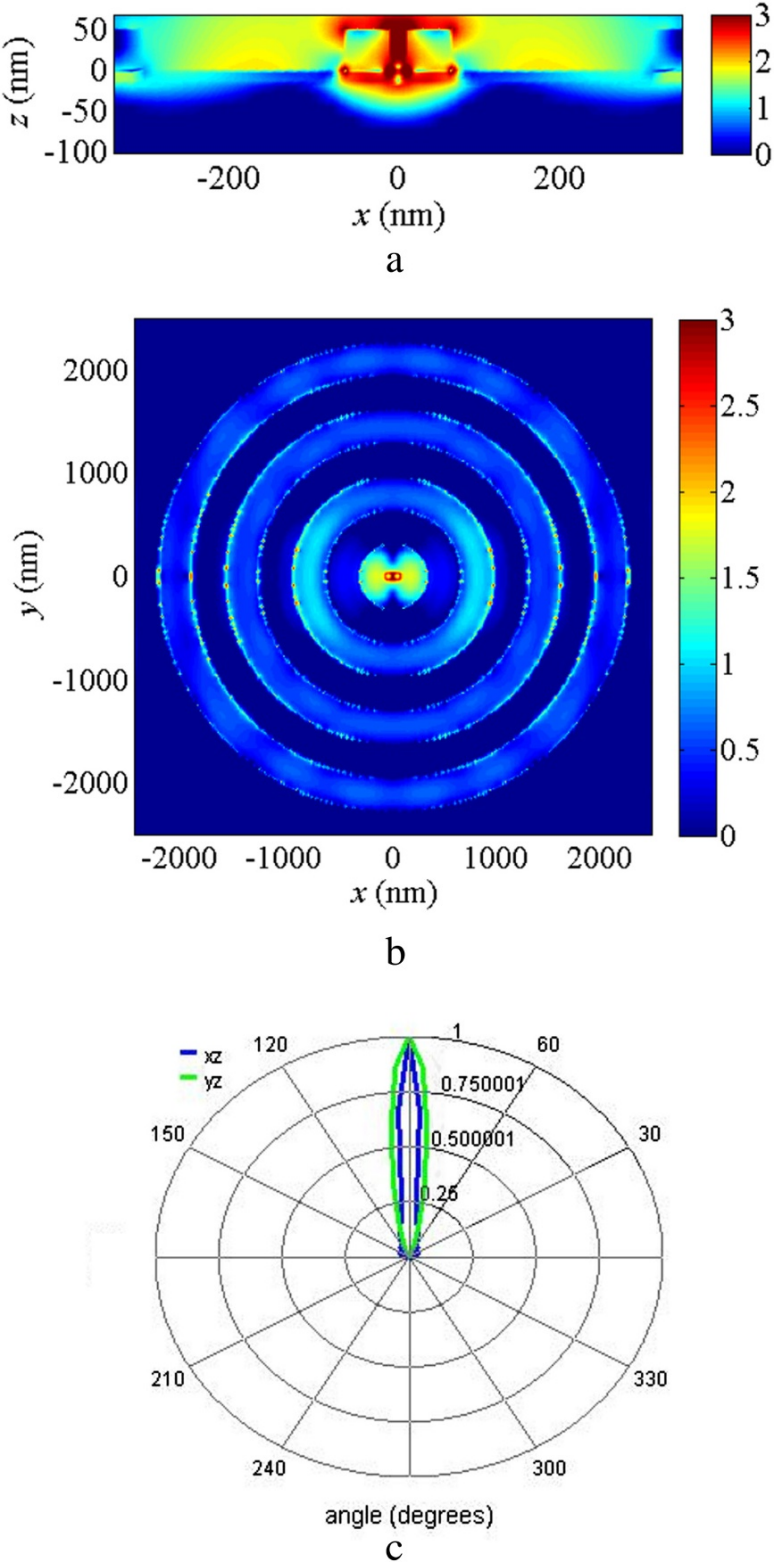
**Near-field intensities and far-field radiation patterns with** ***H*** **= 20 nm.** Near-field intensities and far-field radiation patterns for the structure with dielectric layer thickness 20 nm at *λ* = 671 nm. **(a)** The cross-section view and **(b)** the top view of the electric field distribution of DESR structure at *λ* = 671 nm and *H* = 20 nm in Figure 5. The scale bars are in logarithmic scale and limited to 0 to 3. The maximum field intensity of (a) and (b) are 10^4.30^, 10^4.56^, times of the incident light intensity. The illuminated light is the plane wave propagated from the top to bottom; i.e., in - *z* direction. **(c)** The corresponding far-field radiation pattern which is excited by placing an *x*-polarized electric dipole source at the feed gap of the dipole antenna.

The maximum electric field at the center of the gap appears at *λ* = 671 nm in the case of *H* = 20 nm in Figure 5 and its near-field electric field distributions are shown in Figure 6a,b. The electric field is concentrated in the center region of the hole. The maximum electric field intensity is located at the nanoantenna upper-surface corners, and its enhancement factor is 10^4.6^. Compared to the results in Figure 3c, it is found that the electric field is more concentrated in the nanoantenna in Figure 6a. This is because the dielectric layer thickness is optimized in Figure 6; however, it should be noted that the illuminated light wavelengths are different between the cases in Figures 3 and 6. This phenomena can also be observed in the top view shown in Figure 6b and Figure 3d. It is also found that the light field intensities around the rings in Figure 6b are not as strong as the case in Figure 3d. The far-field radiation for the case of *H* = 20 nm at *λ* = 671 nm by exciting an *x*-polarized electric dipole source which is placed at the feed gap of the dipole antenna is shown in Figure 6c. It has a narrow light beam radiating to the far field. The narrow-beam radiation effect can be explained by an exponentially decaying leaky plasmon wave [19]. Our structure with concentric rings can be viewed as a structure which can support the leaky wave in the outward directions of the nanoantenna. Thus, the narrower beams are observed in Figure 6c and Figure 4b as compared to Figure 4a.

### Sample fabrications and measurements

**Figure 7:**
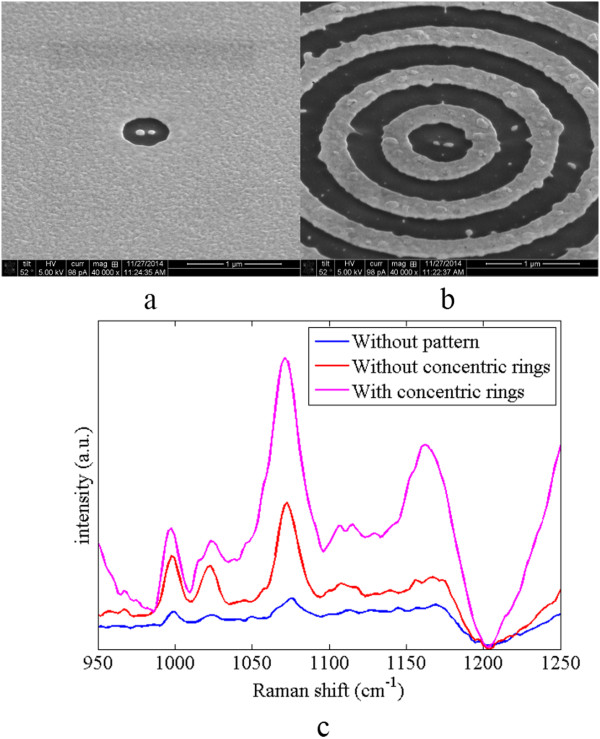
**SEM images and Raman shift spectrum for the structures with and without the concentric rings.** SEM images of **(a)** the DERS structure and **(b)** the structure with concentric rings. **(c)** The Raman shift spectrum of the thiophenol molecule with three different structures, multi-layer film without pattern, structure without concentric rings, and structure with concentric rings.

A 3-nm titanium adhesion layer and a 150-nm gold layer were first deposited onto the SiO_2_ substrate by electron-beam dual gun evaporation. Then, a 40-nm TiO_2_ layer was deposited and a 50-nm gold layer was deposited onto TiO_2_ layer. The patterns with and without concentric rings on the gold layer are melt by FEI Nova 600i dual beam focused ion beam (FEI, Hillsboro, OR, USA). The period of the concentric rings was designed as 650 nm. To cut the 50-nm thickness of gold film, the smallest current 1.5 pA and 30-KV accelerating voltage were applied. Figure 7a,b shows the SEM images of fabricated structures without and with the concentric rings. The gaps between the nanoantennas are about 40.6 nm in both structures. The self-assemble thiophenol was prepared as the target molecule, and the samples were immersed in the thiophenol solution with a concentration of 10^-3^ M for 48 h. A laser beam with *λ* = 785 nm is focused onto the patterns in the microscope with the magnification of objective lens as 50× and numerical aperture as 0.9. Figure 7c is the measured Raman shift spectra of the thiophenol molecule for the structures with and without concentric rings. For comparisons, the Raman shift spectrum for the multi-layer film without any pattern is also shown in Figure 7c. The Raman shifts for the thiophenol molecule are 1,000.1, 1,022.7, and 1,071.4 cm^-1^. The results show that the structure with the concentric rings has stronger Raman signal than the structure without the concentric rings.

## Conclusions

The large field enhancement of our proposed structure can be achieved by optimizing the periodicity of the concentric gold rings, and it is found that it can achieve stronger electric field enhancements as compared to the structure without the concentric rings. The concentric rings can help to couple more light into the nanoantenna over the ground plane. The narrower far-field radiation beam for our proposed structure can be explained by the contributions of the gold rings which can also scatter light into the far field and modify the far-field radiation pattern. The effects of the dielectric layer thickness on the near-field enhancements and far-field radiation are also investigated. It is found that some peaks are strongly dependent on the dielectric material thickness, and the field enhancement of these peaks becomes larger as the dielectric layer thickness is smaller. Compared to DERS structure, our proposed structure not only enhances near-field intensity accompany a better directional far-field pattern in simulation but also has stronger Raman signals. It is useful for some nanoantenna applications for light beam directivity control or strong field enhancement, for example, the sensing or detecting by surface-enhanced Raman scattering.

## Abbreviations

DERS: directivity-enhanced Raman scattering
FDTD: finite-difference time-domain.
